# Effect of Fibre Surface Treatment and Nanofiller Addition on the Mechanical Properties of Flax/PLA Fibre Reinforced Epoxy Hybrid Nanocomposite

**DOI:** 10.3390/polym13213842

**Published:** 2021-11-06

**Authors:** Adnan Amjad, M. Shukur Zainol Abidin, Hassan Alshahrani, Aslina Anjang Ab Rahman

**Affiliations:** 1 School of Aerospace Engineering, Universiti Sains Malaysia, George Town 14300, Malaysia; a.adiamondstar@gmail.com (A.A.); aeshukur@usm.my (M.S.Z.A.); 2 Department of Mechanical Engineering, Najran University, King Abdulaziz Road, P.O. Box 1988, Najran 61441, Saudi Arabia; haalshahrani@nu.edu.sa

**Keywords:** flax/PLA, polymer-matrix composites, hybrid, particle-reinforcement, mechanical properties, electron microscopy, surface treatments

## Abstract

Natural fibre-based materials are gaining popularity in the composites industry, particularly for automotive structural and semi-structural applications, considering the growing interest and awareness towards sustainable product design. Surface treatment and nanofiller addition have become one of the most important aspects of improving natural fibre reinforced polymer composite performance. The novelty of this work is to examine the combined effect of fibre surface treatment with Alumina (Al_2_O_3_) and Magnesia (MgO) nanofillers on the mechanical (tensile, flexural, and impact) behaviour of biotex flax/PLA fibre reinforced epoxy hybrid nanocomposites. Al_2_O_3_ and MgO with a particle size of 50 nm were added in various weight proportions to the epoxy and flax/PLA fibre, and the composite laminates were formed using the vacuum bagging technique. The surface treatment of one set of fibres with a 5% NaOH solution was investigated for its effect on mechanical performance. The results indicate that the surface-treated reinforcement showed superior tensile, flexural, and impact properties compared to the untreated reinforcement. The addition of 3 wt. % nanofiller resulted in the best mechanical properties. SEM morphological images demonstrate various defects, including interfacial behaviour, fibre breakage, fibre pullout, voids, cracks, and agglomeration.

## 1. Introduction

Composite materials are a versatile class of materials with excellent properties and applications. They are made up of two or more chemically distinct constituents, namely the matrix and reinforcement. Typically, the reinforcement is more rigid and superior to the matrix, while the matrix holds the reinforcement and provides a homogenous structure [1]. Although synthetic fibres are the most frequently used reinforcement in polymer composites, growing environmental concerns have recently encouraged the use of renewable resources. Biodegradable reinforcing materials such as natural fibres are the best substitute for synthetic fibres in the composite industry due to their technological and ecological advantages [2]. The mechanical properties and chemical composition of commonly used natural fibres are found in the literature [3,4]. For several applications, including automobiles, construction, and the aerospace industry, research and development (R&D) has proven that natural fibres are advantageous due to their low cost, low density, low CO_2_ emission, nonabrasive nature, low energy consumption, lack of skin irritation, and low health risk [5,6,7,8]. Additionally, natural fibre reinforced polymer composite (NFRPCs) are less expensive, more substantial, and environmentally-friendly [9].

Along with their numerous benefits, natural fibres have a few disadvantages, including incompatibility between the fibre and the matrix, low resistance to moisture, dimensional instability, and a tendency to aggregate during processing [10]. The weak interfacial bond between water-loving natural fibres and a water-repellent polymer matrix results in a decrease in the properties of the polymer composite, which is detrimental for industrial and structural applications [11]. However, numerous schemes and approaches overcome these shortcomings, including chemical treatment of reinforced fibre, hybridization, and filler application [12,13]. Natural fibres chemical composition, surface morphology, and topography are altered when processed with different chemicals. The existing surface treatment methods are alkali, silane, acetylation, benzoylation, peroxide, permanganate, and sodium chlorite [11,14,15,16,17,18]. Alkaline treatment, also known as mercerization, is one of the most widely used and simplest methods for improving the adhesion properties of the fibre matrix. This method modifies the cellulosic structure of natural fibres with sodium hydroxide (NaOH), resulting in increased fibre fragmentation and disaggregation speed. The alkaline treatment removes oil, wax, lignin, and pectin from fibres, resulting in a clean, uniform surface.

Another technique for enhancing the mechanical properties of composite materials is hybridization. Hybrid composites typically contain two or more distinct fibre types within the same matrix, but they also contain a blend of two or more polymers reinforced with the same fibre type. The primary goal of hybridization is to overcome a material’s limitations by supporting it with another material with similar or superior properties to the first [19,20]. Hybridization can create hybrid nanocomposite materials at the nanoscale by combining multiple types of nanofiller or nanomaterial in the same matrix [21]. Due to the unique properties of hybrid nanocomposite materials, they have been used in various applications due to their lower cost than conventional composite materials [22].

The incorporation of nanofillers via advanced manufacturing techniques has improved fibre-reinforced composite materials by modifying the fibre and matrix interaction [23,24]. Nanofillers have a tremendous potential to enhance the mechanical performance of composites, thereby expanding their applications in a myriad of fields. Homogeneous and uniform nanofiller mixing accelerates the composite’s mechanical, thermal, and tribological properties [25,26,27]. Nanofillers are typically inorganic and occasionally organic; the most common inorganic nanofillers are alumina, magnesia, silica, zinc oxide, titanium dioxide, and calcium carbonate; naturally organic nanofillers include synthetic clay, carbon black, and cellulosic fibres [28,29,30]. The optimal quantity of nanofillers varies according to the filler, matrix, and fibre type [31,32,33,34,35,36,37,38,39,40]. By adding the filler, the available free spaces may be reduced, thereby increasing the stiffness of the laminates. The filler can bridge the matrix and the fibre, resulting in increased interaction between them. Once the load is applied, the stress is easily transferred from the polymer matrix to the reinforcing fibre, thus significantly improving their mechanical performance [41]. The addition of nanofillers affects the epoxy matrix’s curing process; this effect depends on the size and concentration of the nanofillers in the matrix. Increased filler content and smaller nanofiller size decrease the curing speed of the epoxy matrix, which may be due to the limited movement space for the polymer chain and monomer in nano filled epoxy composites [42,43,44,45].

Synthetic fibres are toxic, non-disposable, abrasive and involve a complex manufacturing process, while natural fibres are robust, low-cost, and eco-friendly [46,47]. Increasing environmental concerns have lead to a swift change in the research pace from synthetic to natural fibre composite materials [48]. Many previous researchers have reported using natural fibres; however, their results indicate comparatively inferior properties and poor compatibility compared with synthetic composites, which necessitates modification in the fabrication technique [49,50,51]. Nanofiller addition and fibre surface enhancement can be considered effective methods to improve NFRPCs performance [52,53]. However, a notable lack of research exists on this topic. In this study, the combined effect of the fibre surface treatment and the addition of nanofillers was investigated in terms of the mechanical properties of the hybrid nanocomposite. The polymeric composite was manufactured using alkali-treated flax/PLA 2D woven fabric, and nano Al_2_O_3_ and MgO reinforced epoxy composites using the vacuum bagging technique. The effect of surface treatment and nanofiller addition was investigated for tensile, bending and impact properties, and morphology was observed using electron microscopy scanning (SEM).

## 2. Materials and Methods

### 2.1. Materials

The biotex flax/PLA (100% bioderived commingled fabric in which the flax fibre is about 40% and the remaining 60% is PLA) with 2/2 twill weave and 400 g/m^2^ fabric weight with a density of 1.33 g/cm^3^ was obtained from Composite Evolution Limited, Chesterfield, United Kingdom. Infusion epoxy (Pro-set INF-114/INF-213) with a density of 1.14 g/cm^3^ was purchased from Castmech Technologies Sdn. Bhd., Perak, Malaysia. Al_2_O_3_ and MgO nanofillers were supplied by Richest Group Limited, Shanghai, China. The specifications of Al_2_O_3_ and MgO nanofillers are presented in Table 1. Sodium hydroxide (NaOH), Sodium dodecyl sulfate (SDS) as a wetting agent, and Ethylene-diamine-tetra-acetic acid (EDTA) as a sequestering agent were obtained from a local supplier in Malaysia.

### 2.2. Methodology

The factors and their associated levels that were considered in this study are shown in Table 2. The design of experiment (DOE) was generated using the full factorial design technique in the Minitab-19 statistical tool. Thus, a total of eighteen (18) combinations without repetition were formed, nine for each type of reinforcement, treated or untreated.

#### 2.2.1. Alkaline Treatment of 2D Woven Biotex Flax/PLA Fabric

The biotex flax/PLA fabric was pre-treated with a 5% sodium hydroxide (NaOH) solution as in Equation (1) [54,55,56], containing ppm concentrations of sodium dodecyl sulphate (SDS) as a wetting agent that lowers the surface tension of water, allowing drops to spread onto a surface, thereby increasing a liquid’s spreading ability and ethylene-diamine-tetra-acetic acid (EDTA) as a sequestering agent that binds the undesirable metal ions together to form a stable structure. The fabric was then heated to 100 °C for 60 min. After 60 min, the fabric was rinsed with hot water and then with cold water. After washing, the fabric was allowed to air dry for 24 h before being oven-dried for 60 min at 80 °C. After that, treated and untreated biotex flax/PLA fabric was cut into a dimension of (300 mm × 300 mm) to be used as reinforcement.

(1) $$\frac{Flax}{PLA}Fibre-O^{-}H^{+}+Na^{+}-OH^{-}\to \frac{Flax}{PLA}Fibre-O^{-}Na^{+}+H_{2}O$$

#### 2.2.2. Fabrication of Hybrid Composite

The tri-layer hybrid composite with dimensions of 300 mm × 300 mm and a stacking sequence of [0/90/0] was manufactured by vacuum bagging technique using infusion epoxy as the matrix. The three reinforcement layers in all composite samples were of the same type. The mass ratio of fibres to the matrix was 1:2.5. Prior to applying the epoxy, the nanofillers were added in weight percentages (0–4 wt. %) to the epoxy according to DOE, followed by mechanical stirring at a speed of 3000 rpm for 60 min to disperse the nanofillers. After mixing the nanofiller, the mixture was degassed for ten minutes. Then, in a ratio of 100:27.4, the hardener was added to the epoxy. After composite plate fabrication, samples were removed from vacuum bagging after 24 h and placed in an oven for 120 min at a temperature of 80 °C for post-curing of the composite plates.

#### 2.2.3. Characterization

Tensile tests were conducted under the ASTM D3039 standard using the universal testing machine, Instron 3367, equipped with a load cell with a capacity of 30 kN. The gauge was set to a length of 120 mm, and the speed was 2 mm/min. Flexural tests were conducted as per ASTM D7264 standards using a universal testing machine, Testometric M500-50CT, equipped with a 50 kN load cell. The span length was set to 95 mm, and the crosshead speed was 2 mm/min. Impact tests were conducted using the Zwick/Roell^®^ pendulum impact tester following the ISO 179/180 standard. The Charpy method was applied to unnotched samples of each composite type with a hammer impact energy of 7 J. Using a QUANTA FEG 450 scanning electron microscope, the uncoated tensile fracture morphologies of biotex flax/PLA tri-layer hybrid composites were analyzed. The samples were cut into tiny pieces before scanning with a 5 kV accelerating voltage and high vacuum mode. The surface of interest was mounted upright on double-sided electrically conducting carbon adhesive tapes to prevent the specimens from accumulating surface charge during exposure to the electron beam.

## 3. Results

### 3.1. Tensile Properties

**Figure 1:**
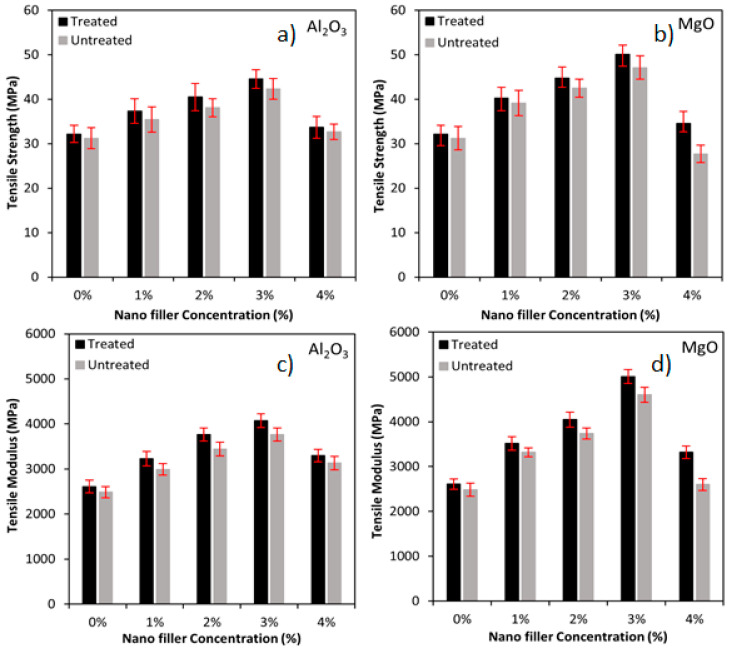
(**a**) Tensile strength of nano Al2O3, (**b**) Tensile strength of MgO, (**c**) Tensile modulus of nano Al2O3, (**d**) Tensile modulus of MgO incorporated flax/PLA epoxy hybrid composites.

The tensile strength and modulus of the treated and untreated biotex flax/PLA fibre reinforced hybrid nanocomposite with nano Al_2_O_3_ and MgO are depicted in Figure 1. It is revealed that the addition of nano Al_2_O_3_ and MgO to the treated and untreated flax/PLA fibre reinforced hybrid composites increases tensile strength and modulus by transmitting and distributing the applied load. Adding the filler may reduce the available free spaces and hence increase the stiffness of the laminates. The filler can link the matrix and the fibre, leading to enhanced interaction between the fibre and the matrix. Once the load has been applied, the stress can easily be transferred from the polymer matrix to the reinforcing fibre, thus improving the tensile strength and modulus of the hybrid composite [31,52,57].

Additionally, the Al_2_O_3_ and MgO nanofiller in the biotex flax/PLA epoxy hybrid nanocomposites exhibit similar tensile strength and modulus behaviour. Biotex flax/PLA reinforcement that has been treated outperforms the untreated version because the sodium hydroxide (NaOH) modifies the flax/PLA cellulosic structure, which increases fibre fragmentation and disaggregation. The alkaline treatment removed the oil, wax, lignin, and pectin from fibres. The surface becomes clean and free from dirt and impurities with functional moieties, helping improve the interfacial bonding between fibres and matrix [11,18]. As illustrated in Figure 1a,c, the tensile strength of the treated biotex flax/PLA in the presence of nano-Al_2_O_3_ (1–3%) increased by approximately 16%, 26% and 38% as compared to untreated, which has 14%, 22% and 35%, respectively. The modulus of the treated reinforcement was increased by 24%, 44% and 56% compared to untreated, which has 20%, 39% and 52%, respectively, concerning the sample with 0% of nano-Al_2_O_3_. Further increase in the nanofiller concentration to 4% had reduced the tensile strength by 24% (treated) and 23% (untreated), and modulus by 19% (treated) and 17% (untreated), respectively, when compared to the sample with 3% of nano-Al_2_O_3_. The decrease is primarily due to a poor fibre-matrix interface; rather than uniformly dispersing, the nanofillers agglomerate, reducing the surface area and bonding between fibre and matrix, both of which contribute to the crack initiation [1,52,58]. The acquired results are also in agreement with other reported research involving short nylon fibre/nanoclay [59], coir, wood fibre/nanoclay/PP [60], and carbon/graphene/epoxy [61].

In the case of nano-MgO, the tensile strength and modulus of the treated biotex flax/PLA in the presence of nano-MgO (1–3%) increased by approximately 25%, 39% and 56% as compared to untreated, which has 25%, 36% and 51%, respectively, while the modulus of the treated reinforcement increased by 35%, 55% and 92% as compared to untreated which has 34%, 50% and 85%, respectively, when compared to the unfilled sample (0% of nano-MgO). Further increase in the nanofiller concentration to 4% reduced the tensile strength by 31% (treated) and 41% (untreated) and the modulus by 34% (treated) and 44% (untreated), respectively, when compared to the sample with 3% of nano-MgO as shown in Figure 1b,d. The tensile strength and modulus of Al_2_O_3_-filled hybrid nanocomposite are higher than those of MgO-filled nanocomposite. This could be due to the higher molecular weight and higher topological polar surface area of Al_2_O_3_ nanofillers. A similar conclusion was reported in luffa/PbO/epoxy [52], short nylon fiber/nanoclay [59], coir, wood fiber/nanoclay/PP [60], and carbon/graphene/epoxy [61].

**Figure 2:**
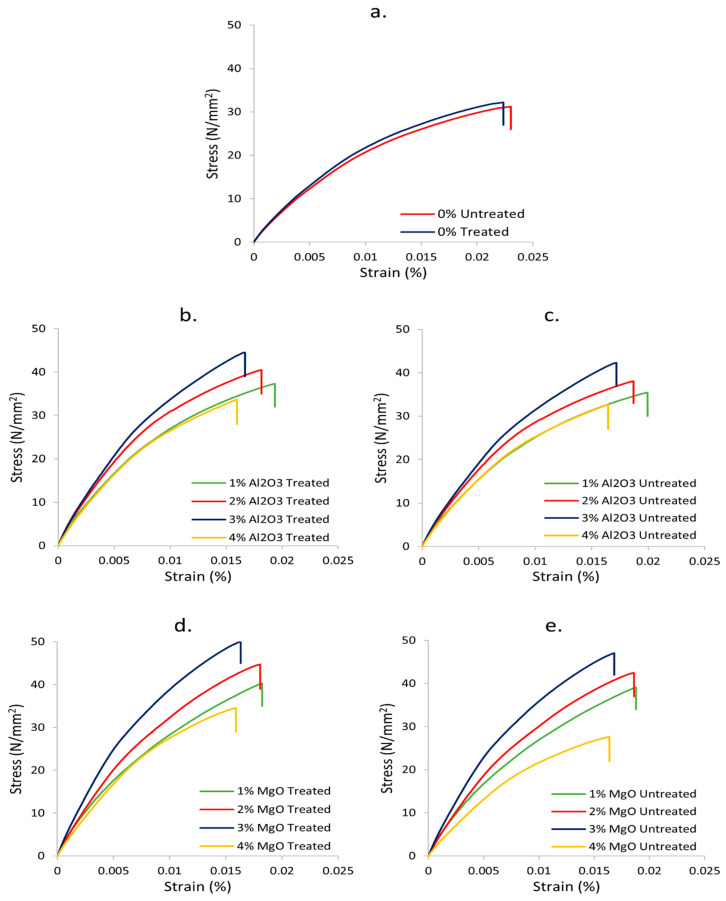
Tensile stress–strain curves of (**a**) neat composite, (**b**) Al_2_O_3_—treated, (**c**) Al_2_O_3_—untreated, (**d**) MgO—treated, and (**e**) MgO—untreated flax/PLA epoxy hybrid composites.

The tensile stress–strain curves of treated and untreated biotex flax/PLA epoxy hybrid nanocomposite with nanofiller Al_2_O_3_ and MgO are shown in Figure 2. Tensile stress–strain curves demonstrate that as the nanofiller concentration increases from 1% to 4%, the strain percentage decreases gradually, which indicates that the brittleness of the composite increases. This decrease in strain can be attributed to restriction of the movement of molecular chains by the nanofillers resulting in improved tensile properties [13]. Additionally, it noted that none of the samples failed abruptly, but exhibited pseudo-plastic behaviour before reaching the ultimate tensile stress value.

The increasing trend in tensile strength and modulus can be explained by the large surface area of the nanofillers, which act as a link between the biotex flax/PLA fibre and epoxy matrix, resulting in improved bonding. Thus, the applied load can be easily transferred from matrix to fibre, resulting in delayed crack initiation. Therefore, it can be concluded that the incorporation of nanofillers increases interfacial adhesion, resulting in an increase in tensile properties, which is also reported in previous studies [1,52,58].

### 3.2. Flexural Properties

**Figure 3:**
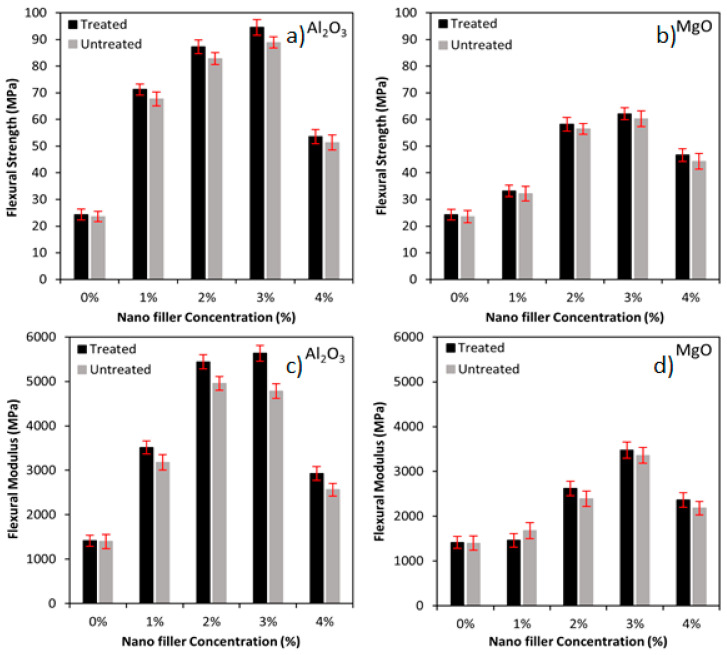
(**a**) Flexural strength of nano Al_2_O_3_, (**b**) Flexural strength of MgO, (**c**) Flexural modulus of nano Al_2_O_3_, (**d**) Flexural modulus of MgO incorporated flax/PLA epoxy hybrid composites.

The flexural strength and modulus of the treated and untreated biotex flax/PLA fibre reinforced hybrid nanocomposite with Al_2_O_3_ and MgO nanofiller are illustrated in Figure 3. It can be seen that the addition of Al_2_O_3_ and MgO nanofillers to treated and untreated flax/PLA fibre reinforced composites increase flexural strength and modulus via interfacial adhesion between the nanofillers and epoxy matrix. The surface energy and rigidity of the nanofillers dictate the extent to which they interact with the matrix. Nanofillers such as Al_2_O_3_ and MgO have higher surface energy and rigidity, which results in a more robust interaction and thus a higher flexural strength and modulus of the filled composite, at the expense of ductility [58].

Additionally, the Al_2_O_3_ and MgO nanofiller in the biotex flax/PLA epoxy hybrid nanocomposites exhibit similar flexural strength and modulus behaviour. Biotex flax/PLA reinforcement that has been treated outperforms untreated one. As illustrated in Figure 3a,c, the flexural strength of the treated biotex flax/PLA in the presence of nano-Al_2_O_3_ (1–3%) increased by approximately 193%, 259% and 289% as compared to untreated, which has 187%, 252% and 277%, respectively. In contrast, the modulus of the treated reinforcement was increased by 148%, 285% and 298% compared to untreated, which has 227%, 254% and 242%, respectively, when compared with unfilled samples (0% of nano-Al_2_O_3_). Further increase in the nanofiller concentration to 4% reduced the flexural strength by 43% (treated) and 42% (untreated) and the modulus by 48% (treated) and 46% (untreated), respectively when compared with the sample with 3% of nano-Al_2_O_3_. This lowering is due to nano-Al_2_O_3_ aggregation, detrimental to the fabricated nanocomposites’ physical and mechanical properties. The greater decrease in flexural strength and modulus for treated samples was due to better fibre fragmentation and disaggregation by alkali treatment, which produces more active sites on the fibre surface where the nanofillers have higher attraction. Similar results are reported in glass/SiO_2_/epoxy [31] and luffa/PbO/epoxy [52].

In the case of nano-MgO, the flexural strength and modulus of the treated biotex flax/PLA in the presence of nano-MgO (1–3%) increased by approximately 36%, 140% and 156% as compared to untreated, which has 36%, 140% and 156%, respectively. The modulus of the treated reinforcement was increased by 3%, 85% and 146% as compared to untreated, which has 20%, 71% and 140%, respectively compared to the neat sample (0% of nano-MgO). Further increase in the nanofiller concentration to 4% reduced the flexural strength by 25% (treated) and 26% (untreated), and the modulus by 32% (treated) and 35% (untreated), respectively when compared with the sample with 3% of nano-MgO, as shown in Figure 3b,d. This decrease is due to nano-MgO aggregation, which negatively impacts the mechanical and physical properties of the fabricated nanocomposites, with similar results reported in glass/SiO_2_/epoxy [31] and luffa/PbO/epoxy [52].

**Figure 4:**
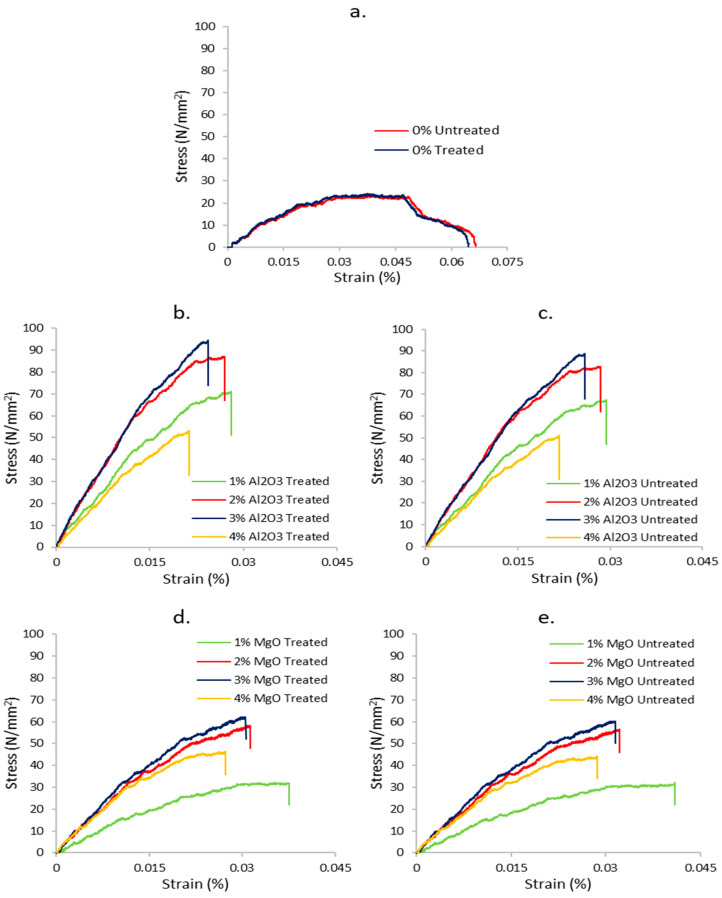
Flexural stress–strain curves of (**a**) neat composite, (**b**) Al_2_O_3_—treated, (**c**) Al_2_O_3_—untreated, (**d**) MgO—treated, and (**e**) MgO—untreated flax/PLA epoxy hybrid composites.

The flexural stress–strain curves of treated and untreated biotex flax/PLA epoxy hybrid nanocomposite with nano Al_2_O_3_ and MgO are shown in Figure 4. Flexural stress–strain curves demonstrate that as the nanofiller concentration increases from 1% to 4%, the strain percentage decreases gradually, which indicates that the ductility of the composite decreases. This is associated with less chain mobility and deformability of the matrix due to the presence of additional rigid nanofillers, which disturb the deformation of the crystalline region in the matrix, resulting in improved bending properties [13,62]. Additionally, it is noted that none of the samples failed abruptly, but exhibited pseudo-plastic behaviour before reaching the ultimate flexural stress value. Flexural strength follows a similar trend to tensile strength, although it varies less between formulations than tensile strength [36].

In Figure 4, there is a slight change in the stress–strain values of the treated and untreated fibre reinforced composite, similar to the tensile stress and strain. The flax/PLA is 100% bioderived commingled fabric in which the percentage of flax fibre is 40% and the remaining 60% is PLA; thus, the alkali treatment only alters the surface properties of the natural fibres, which is responsible for the reduced difference between the treated and untreated reinforcement.

### 3.3. Impact Properties

**Figure 5:**
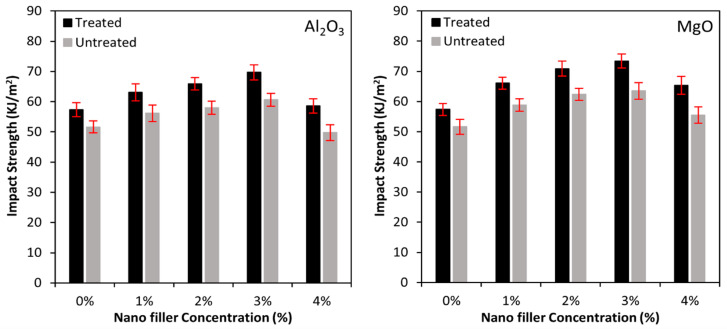
Impact strength of nano Al_2_O_3_ and MgO incorporated biotex flax/PLA epoxy hybrid composites.

The unnotched impact strength of biotex flax/PLA fibre (treated and untreated) and nanofiller reinforced epoxy composite is illustrated in Figure 5. Charpy impact strength (calculated by dividing the absorbed energy with sample width and thickness) is related to the material’s toughness, and the fibre-matrix interface and bonding between the lamina indicated the nanocomposite’s fracture toughness. Nanofillers such as Al_2_O_3_ and MgO improved the bonding between the filler and matrix, and their rigidity increased energy absorption. Energy absorption in polymer nanocomposites occurs due to debonding at the fibre–matrix interface, the fracturing of the reinforcing material, and matrix plastic deformation [4,58].

Additionally, the Al_2_O_3_ and MgO nanofiller in biotex flax/PLA epoxy hybrid nanocomposite exhibit similar impact properties as tensile and flexural properties. Treated biotex flax/PLA reinforcement outperformed as compared to the untreated. The impact strength of the treated biotex flax/PLA in the presence of nano-Al_2_O_3_ (1–3%) increased approximately by 10%, 15% and 22% as compared to untreated, which has 9%, 12% and 17%, respectively, as compared to unfilled sample (0% of nano-Al_2_O_3_). Further increase in the nanofiller concentration to 4% reduced the impact strength by 16% (treated) and 18% (untreated) in the sample with 3% of nano-Al_2_O_3_. Similar results are observed for a research study on bagasse/nanoclay/PP [63].

In the case of nano-MgO, the impact strength of the treated biotex flax/PLA in the presence of nano-MgO (1–3%) increased by approximately 15%, 24% and 28% as compared to untreated, which has 14%, 21% and 23%, respectively, as compared to the neat sample (0% of nano-MgO). Furthermore, an increase in the nanofiller concentration to 4% decreased the impact strength by 11% (treated) and 13% (untreated), concerning the sample having 3% nano-MgO. The presence of Al_2_O_3_ and MgO nanofiller in the epoxy matrix generates matrix discontinuity, which provides sites for crack initiation, resulting in decreased impact strength at 4% nanofillers; similar results are reported in bagasse/SiO_2_/PE [37] and PLA/nanoclay/LLDE [64].

### 3.4. Scanning Electron Microscope (SEM) Failure Analysis

**Figure 6:**
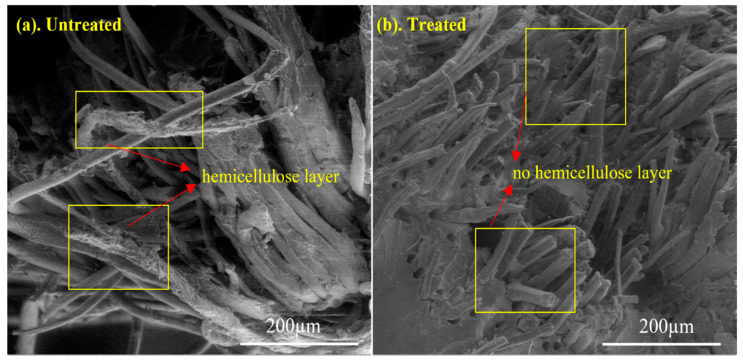
SEM images of tensile fractured (**a**) Untreated (**b**) alkali-treated biotex Flax/PLA reinforced epoxy composite.

SEM analysis was used to characterize the surface characteristics of the tensile broken composite material utilized in this investigation. The SEM micrographs of treated and untreated biotex flax/PLA reinforced epoxy composites without nanofillers are shown in Figure 6 at 500× magnification. The literature shows that the alkaline treatment of natural fibre removed the hemicellulose layer from the surface of fibres [19,52]. In Fourier transform infrared spectroscopy (FTIR) analysis, the disappearance of the C=O stretching vibration of hemicellulose at 1750 cm^−1^ indicates the removal of hemicellulose that strengthens the fibre matrix interface, and results in improved mechanical properties of composites [53,65]. This finding is further validated by Figure 6, which shows that the alkali treatments removed impurities from the fibre surface, such as hemicelluloses and lignin. This uneven topography aided mechanical interlocking. It also improved fibre–matrix adhesion, which improved mechanical qualities. In the same way, a corresponding result was accomplished with jute/epoxy composite [65,66,67].

**Figure 7:**
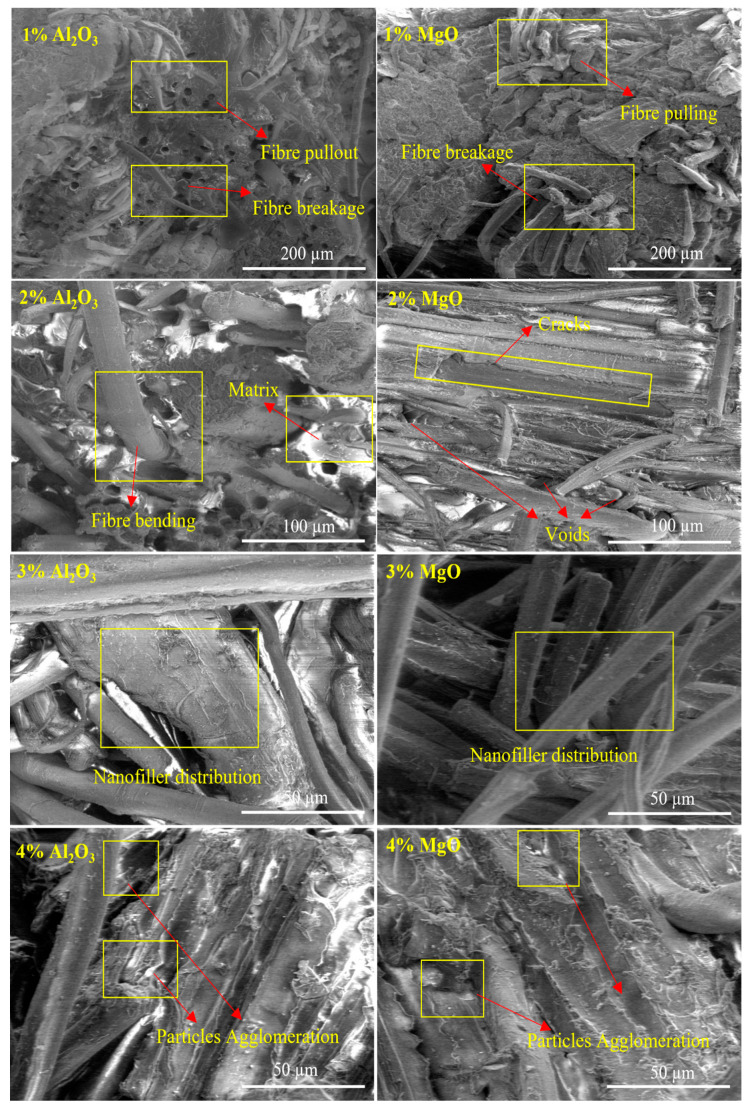
SEM images of alkali-treated biotex flax/PLA reinforced epoxy composite at different percentage of nano-Al_2_O_3_ and nano-MgO.

**Figure 8:**
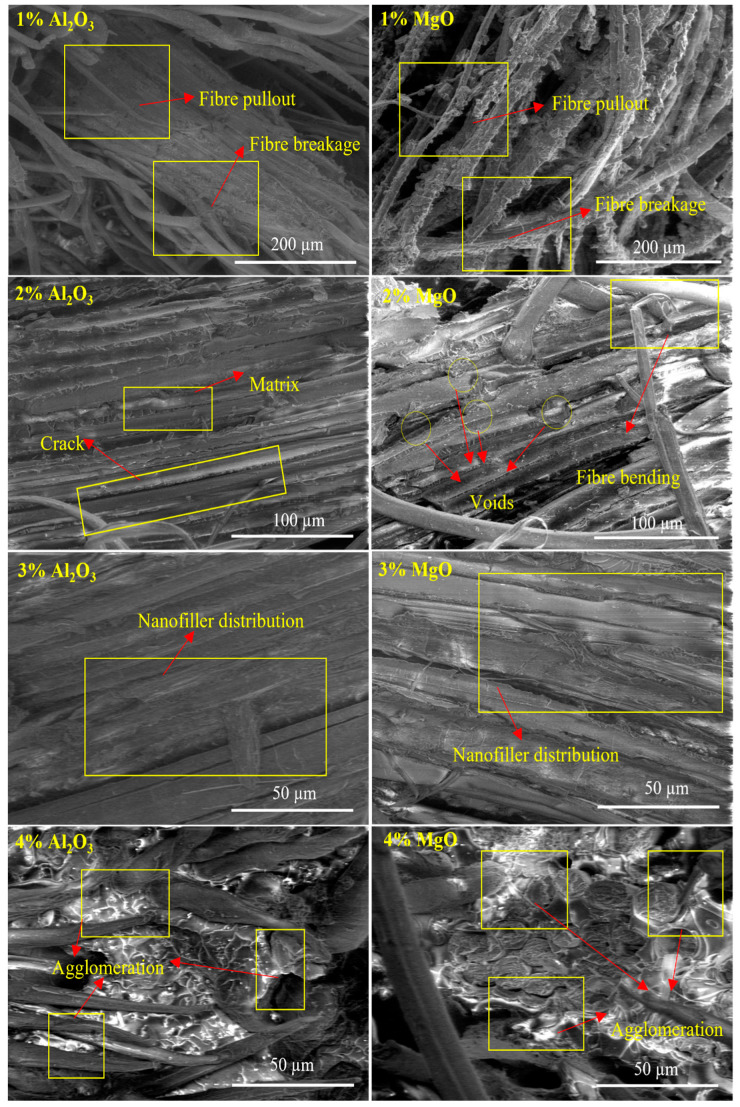
SEM images of untreated biotex flax/PLA reinforced epoxy composite at different percentages of nano-Al_2_O_3_ and nano-MgO.

Figure 7 and Figure 8 show SEM micrographs of biotex flax/PLA reinforced epoxy hybrid nanocomposites with Al_2_O_3_ and MgO nanofiller. The fractured surface of the tensile samples was analyzed to determine fibre failure and matrix–fibre interfacial bonding. Micrograph images of treated biotex flax/PLA reinforced epoxy composites with nanofiller Al_2_O_3_ and MgO at concentrations ranging from 1% to 4% are shown in Figure 7. The micrograph images indicate that the strength of the polymer composite is highly dependent on the fibre–matrix interface bonding. The fibre–matrix interface of the composite with 1% filler exhibits severe fibre pullout, fibre breakage, cracks, and air bubbles, resulting in poor bonding, low strength, and a poor fibre–matrix interface.

However, this interface is better than that of the neat composite. As the amount of nanofiller in the matrix increases, the fibre–matrix interface improves. Better fibre–matrix bonding was observed when the matrix contained 2% and 3% nanofiller. Minimum fibre pullout indicates improved fibre–matrix interfacial adhesion; this aids in transferring load from the matrix to the fibre, thereby increasing its strength. The decrease in mechanical properties caused by the aggregation of nanofiller at 4% is also justified by the micrograph images, which restrict the interaction of the nanofiller with the matrix, resulting in the laminates’ poor mechanical properties. The same trend was observed in micrograph images (as shown in Figure 8) of untreated biotex flax/PLA reinforced epoxy composites with nanofiller Al_2_O_3_ and MgO at concentrations ranging from 1% to 4%. Similar SEM images are also analyzed for oil palm nanofiller/kenaf fibre/epoxy hybrid nanocomposites [1] and lead oxide nanofiller/luffa fibre/epoxy hybrid nanocomposites [52].

## 4. Conclusions

The purpose of this study was to develop a novel hybrid nanocomposite by incorporating nano-Al_2_O_3_ and nano-MgO as reinforcing fillers into biotex flax/PLA fibre mat reinforced epoxy composites. The results of this study are encouraging, as the hybrid nanocomposites fabricated using Al_2_O_3_ and MgO nanofiller and flax/PLA fibres exhibit improved mechanical properties. The addition of nanofiller in the alkali-treated biotex flax/PLA/epoxy composites significantly enhances the mechanical strength in tensile, bending, and impact strength compared to the untreated biotex flax/PLA reinforced epoxy composites, by hindering the crack initiation or propagation paths. Morphological analysis using SEM demonstrated that adding 3% nano-Al_2_O_3_ and 3% nano-MgO filler to flax/PLA/epoxy composites reduces void contents, fibre pull out, fibre protrusion, and tearing on the fractured surface by simply fracturing or breaking/bending the fibre due to the fibre’s improved adhesion and interfacial bonding with the matrix. The enhanced mechanical and morphological properties of the nano-(Al_2_O_3_ & MgO)/(flax/PLA)/epoxy hybrid nanocomposites indicate a high potential for construction and structural applications requiring renewable resources high performance. As a result of their superior morphological and mechanical properties, the developed hybrid nanocomposites will serve as a low-cost, lightweight, and environmentally friendly composite material for use as a building material. This research could be expanded in the near future by varying types, sizes, and concentrations of nanofillers and different natural fibres and chemical treatment methods. 

## Figures and Tables

**Table 1:** Specifications of Al_2_O_3_ and MgO nanofillers.

Properties	Al_2_O_3_	MgO
Size (nm)	50 nm	50 nm
Shape	Spherical	Spherical
Colour	White	White
Purity (%)	99%	99%
Density (g/cm^3^)	3.95 g/cm^3^	3.58 g/cm^3^

**Table 2:** Factors and levels.

Factors	Levels					
Reinforcement	Treated flax/PLA			Untreated flax/PLA		
Nanofiller Type	Al_2_O_3_			MgO		
Nanofillers Concentration (wt. %)	0	1	2		3	4

## Data Availability

The data presented in this study are available on request from the corresponding author. The data are not publicly available due to privacy of this research.
